# Sequence features associated with microRNA strand selection in humans and flies

**DOI:** 10.1186/1471-2164-10-413

**Published:** 2009-09-04

**Authors:** Hai Yang Hu, Zheng Yan, Ying Xu, Hao Hu, Corinna Menzel, Yan Hong Zhou, Wei Chen, Philipp Khaitovich

**Affiliations:** 1 Partner Institute for Computational Biology, 320 Yue Yang Road, Shanghai, 200031, PR China; 2 Hubei Bioinformatics and Molecular Imaging Key Laboratory, 1037 Luoyu Road, Wuhan, 430074, PR China; 3 Max Planck Institute for Molecular Genetics, Ihnestrasse 63-73, D-14195 Berlin, Germany; 4 Max Delbrück Center for Molecular Medicine, Robert-Rössle-Strasse 10, 13125 Berlin, Germany; 5 Max Planck Institute for Evolutionary Anthropology, Deutscher Platz 6, D-04103 Leipzig, Germany

## Abstract

**Background:**

During microRNA (miRNA) maturation in humans and flies, Drosha and Dicer cut the precursor transcript, thereby producing a short RNA duplex. One strand of this duplex becomes a functional component of the RNA-Induced Silencing Complex (RISC), while the other is eliminated. While thermodynamic asymmetry of the duplex ends appears to play a decisive role in the strand selection process, the details of the selection mechanism are not yet understood.

**Results:**

Here, we assess miRNA strand selection bias in humans and fruit flies by analyzing the sequence composition and relative expression levels of the two strands of the precursor duplex in these species. We find that the sequence elements associated with preferential miRNA strand selection and/or rejection differ between the two species. Further, we identify another feature that distinguishes human and fly miRNA processing machinery: the relative accuracy of the Drosha and Dicer enzymes.

**Conclusion:**

Our result provides clues to the mechanistic aspects of miRNA strand selection in humans and other mammals. Further, it indicates that human and fly miRNA processing pathways are more distinct than currently recognized. Finally, the observed strand selection determinants are instrumental in the rational design of efficient miRNA-based expression regulators.

## Background

MicroRNAs are small single-stranded endogenous RNAs, approximately 22 nucleotides in length, which are involved in posttranscriptional gene regulation in a wide variety of species [1-4]. miRNAs function as a component of an RNA-Induced Silencing Complex (RISC) by guiding it to specific targets through base-pairing interaction between the miRNA seed region and a complementary sequence in the 3'-UTR of a target transcript [5,6]. In humans and other animals, the seed region normally extends from the second to eighth positions of mature miRNA [7].

All known miRNAs originate from a single-stranded RNA precursor shaped as a hairpin loop structure [8,9]. In animals, the hairpin is excised from a longer precursor by Drosha and subsequently cut by Dicer, which produces an RNA duplex with 3' overhanging ends, each 2 nucleotides long [8,10-13]. This duplex structure is shared between miRNA and small interfering RNA (siRNA) processing pathways [14,15]. For both miRNAs and siRNAs, one of the duplex strands is then incorporated into the RISC and the other eliminated. Since the selected strand determines the functional specificity of an RISC, this is a crucial step in the RNA interference (RNAi) pathway.

Commonly, the two strands are incorporated into an RISC with different probabilities [16]. In siRNAs, strand selection probability depends on the relative thermodynamic stability of the ends of the precursor duplex: a strand with lower duplex stability at the 5'-end is preferentially selected [14,16]. A single mismatch within the first four nucleotides of an siRNA duplex is sufficient to determine strand selection specificity. Further, a single nucleotide substitution at the 5'-end of an siRNA that changes thermodynamic asymmetry of a duplex is sufficient to completely reverse it [14]. In flies, the thermodynamic asymmetry of the siRNA duplex is recognized by preferential binding of the Dcr-2/R2D2 protein heterodimer to its more stable end, thus promoting asymmetric strand loading into the Ago2-RISC complex ([17]. Importantly, however, most fly miRNAs are loaded into a different silencing complex, Ago1-RISC, by an unknown mechanism independent of Dcr-2/R2D2 [18]. Thus, the strand selection determinants used in fly miRNA and siRNA pathways may differ.

Sequence analysis of 16 fly (*Drosophila melanogaster*), 96 worm (*Caenorhabditis elegans*), 73 mouse, and 87 human miRNAs has shown that their miRNA precursors exhibit a thermodynamic stability bias similar to that of siRNAs, *i.e. *the strand with the less stable 5'-end is preferentially represented [16]. Based on this observation, it was proposed that miRNAs and siRNAs might share the same strand selection determinants [16]. Recent analysis of human and mouse miRNA expression profiles, however, has shown that the relative expression levels of the two strands vary widely among tissues [19]. Notably, in some tissues tested, miRNAs originating from a thermodynamically unfavourable strand are present at levels comparable to or greater than that of their thermodynamically favourable counterparts.

In this study, we determine sequence elements associated with preferential miRNA strand selection and/or rejection by analyzing the sequence composition and relative expression levels of miRNA pairs originating from two strands of the same precursor duplex in high-throughput sequencing data from humans and flies.

## Results and Discussion

### Single precursor commonly generates two mature miRNAs

**Figure 1:**
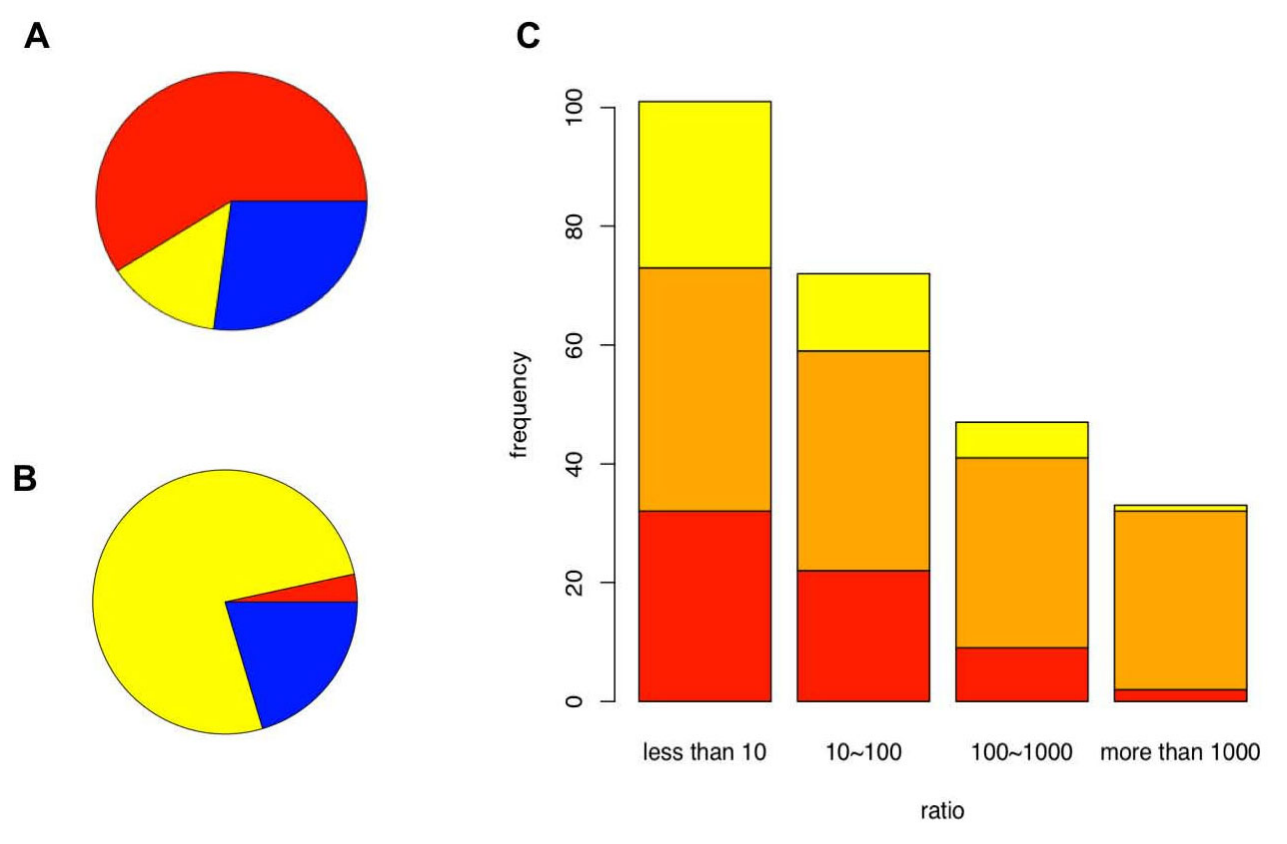
**Expression of miRNA pairs originating from the same precursor**. (**A**) Number of mature miRNA types generated per precursor in humans. A total of 347 known human precursors are represented in the dataset by at least one mature miRNA. Shown are the proportions of miRNA precursors annotated to generate two mature miRNAs (red), annotated to generate one mature miRNA, but generating two in our dataset (yellow), annotated to generate one and generating one mature miRNA in our dataset (blue). (**B**) Number of mature miRNA types generated per precursor in fruit fly. The total number of precursors shown is 147 and the colour code is the same as for human precursors. (**C**) Human miRNA precursors sorted according to the expression ratio between high- and low-expressed miRNA strands originating from the same precursor duplex. The colours represent miRNA pairs annotated as miRNA/miRNA* (orange, *N *= 140), as miRNA 5p/3p (red, *N *= 65), and the novel miRNA pairs (yellow, *N *= 48) discovered in our dataset.

To quantify human miRNA expression levels, we isolated total small RNAs (ranging in size from 18 to 30 nucleotides) from the cerebral cortex of 20 healthy adult human males and sequenced the pooled sample using Illumina high throughput sequencing technology. We then mapped the resulting 6,368,000 sequence reads from a single sequencing lane to the human genome (see Methods). Of these sequences, 3,717,948 map to 449 annotated human miRNAs with a sequence copy number per miRNA ranging from two to 1,248,918 (see Methods). As reported previously, the majority of miRNAs (320 or 71.3% in our data) originate from precursors (pre-miRNA) containing two annotated miRNAs [19] (see Figure 1A). Further, for 48 of the remaining 129 precursors with one annotated miRNA, we find at least two sequence reads that correspond to their unannotated miRNA counterparts (see Methods). Thus, by examining data from a single Illumina sequencing lane, we can substantially reduce the number of miRNA precursors that generate one expressed mature miRNA. Given that mature miRNAs originate from a double-stranded precursor, it is likely that sufficient sequencing efforts will uncover the miRNA counterparts of all known miRNAs.

Although each miRNA precursor can potentially produce two mature miRNAs, these miRNAs are not expressed in a tissue at the same level. In fact, we find no correlation between expression levels of two miRNAs originating from the same precursor in our data, indicating that relative strand abundance within miRNA pairs differs widely among precursors (Pearson correlation, *R *= -0.02, *p *= 0.70, *N *= 253) (see Additional file 1: Figure S1). This absence of any correlation within miRNA pairs starkly contrasts with the strong positive correlation observed between miRNA expression measurements in technical and biological replicates (*R *= 0.97, *p *< 2.2e-16, *N *= 497 and *R *= 0.96, *p *< 2.2e-16, *N *= 467, respectively) (see Methods and Additional file 1: Figure S1). Further, excluding 52 miRNA precursors known to undergo posttranscriptional regulation [20-22] or posttranscriptional editing [23,24] did not affect the result (*R *= 0.0152, *p *= 0.82).

To investigate the pattern of expression differences within miRNA pairs further, we determined expression ratios between high- and low-expressed miRNAs in all 253 pairs, including precursors with one of the two annotated miRNAs not detected in our dataset, but annotated in the miRBase. We find a broad range of differences, with an expression ratio greater than 1000 for 33 miRNA pairs, and less than 10 for 103 miRNA pairs (see Figures 1C and Additional file 1: Figure S2). The miR-let-7f precursor shows the largest difference between high- and low-expressed miRNAs: 1,248,918 copies and three copies, respectively.

Notably, in agreement with previous observations [19], although the observed expression level differences agree somewhat with the existing annotation of miRNA pairs as 5p/3p or miRNA/miRNA*, many deviations occur. In addition, considering high expression of some miRNAs* [25], the classification of all miRNAs originating from the same precursor as 5p and 3p miRNAs proposed in [19] might be more forthcoming than the current one.

### Strand selection determinants of human miRNAs

**Figure 2:**
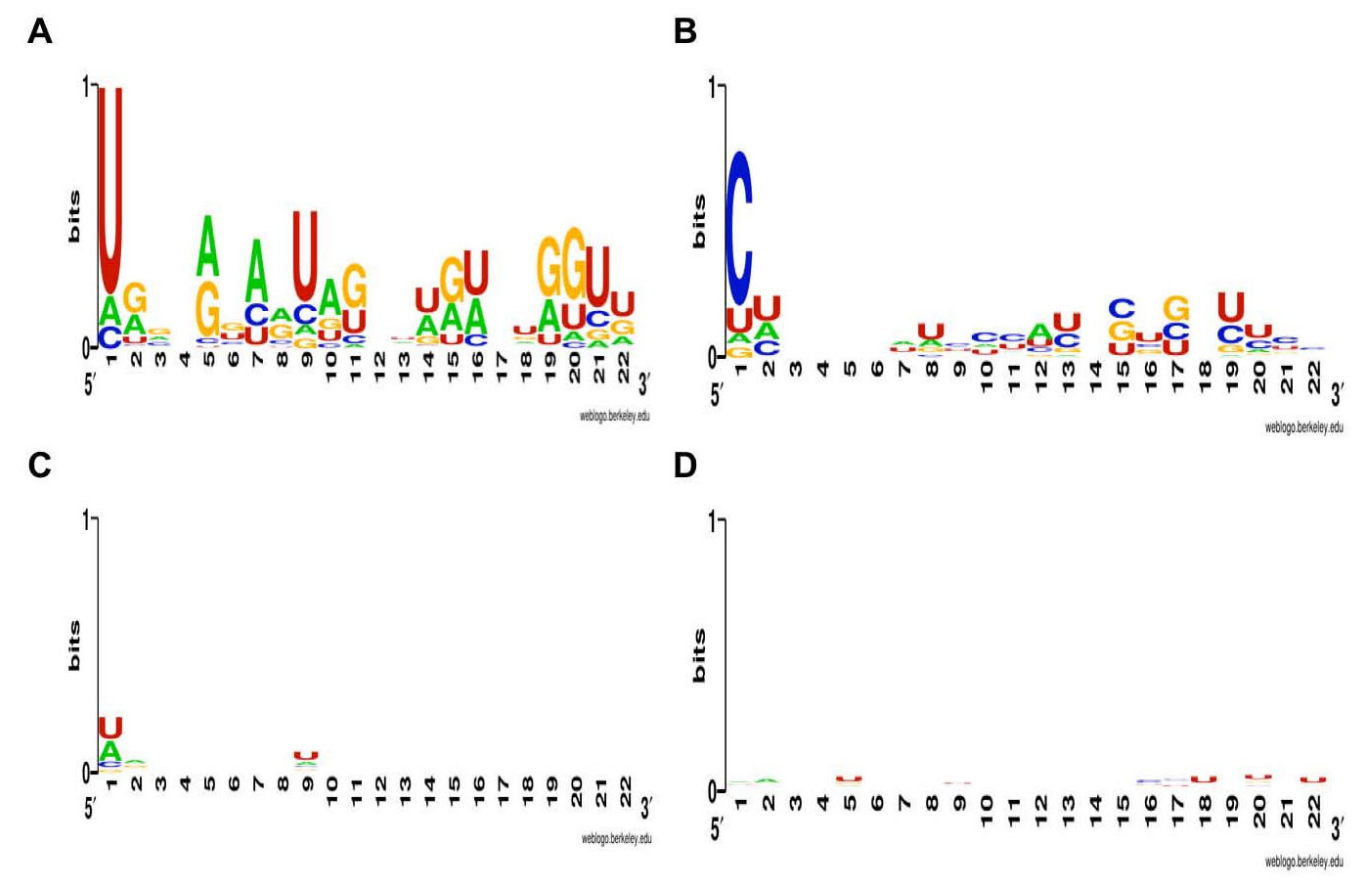
**Sequence features of human miRNA strand selection: 5' end nucleotide**. Sequence logos showing nucleotide composition of high-expressed strand (**A**) and low-expressed strand (**B**) from 33 miRNA pairs with large strand selection bias, and from 103 miRNA pairs with little bias: high-expressed (**C**) and low-expressed (**D**).

**Figure 3:**
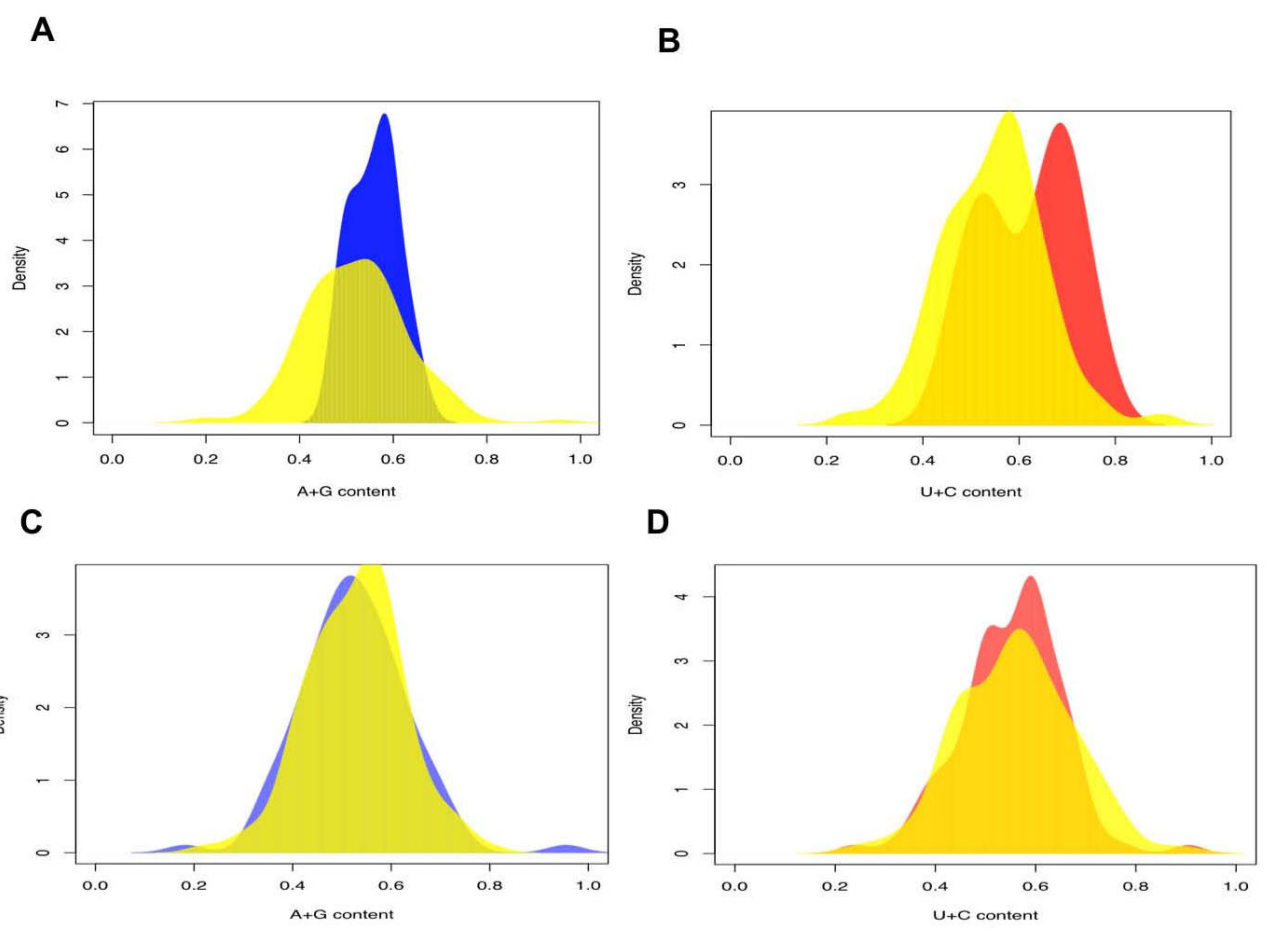
**Sequence features of human miRNA strand selection: purine/pyrimidine bias**. (**A**) Purine content of the high-expressed strand from 33 miRNA pairs with large strand selection bias (blue) and the high-expressed strand from all other expressed miRNA pairs (yellow). (**B**) Pyrimidine content of the low-expressed strand from 103 miRNA pairs with little strand selection bias (red) and the low-expressed strand from all other expressed miRNA pairs (yellow). Purine (**C**) and pyrimidine (**D**) content of high- and low-expressed strands from 103 miRNA pairs with little strand selection bias. Colour labels as on panels (**A**) and (**B**).

Expression level differences between two miRNAs originating from the same precursor cannot result from differential transcription efficiency; therefore, they must be caused by differential stability due to biased strand incorporation into the RISC. To test whether particular sequence features may explain this bias, we compared the sequence composition of the 33 miRNA pairs that exhibit an expression difference greater than 1000-fold between high- and low-expressed duplex strands to all 847 annotated human miRNAs, as well as the 48 new miRNAs identified in this study (see Additional file 1: Table S1). We find three characteristic sequence features: (*i*) a U-bias at the 5'-end of the highly expressed strand (One-sided Binomial test, *p *= 7.9*10^-08^); (*ii*) a C-bias at the 5'-end of the low expressed strand (One-sided Binomial test, *p *= 4.2*10^-12^); (*iii*) an excess of purines (A and G) in the highly expressed strand and, consequently, an excess of pyrimidines (U and C) in the low expressed strand (One-sided Wilcoxon test, *p *< 0.02 in all comparisons) (see Table 1, Figures 2 and 3).

**Table 1:** Sequence features associated with strand selection in human and fly miRNAs

			**Human**	**Fly**
All annotated miRNA and new microRNA*		First nuclotide	U (36%)	U (51%)
		Purine or Pyrimidine bias	No	No

Strand-biased miRNA	Selected strand	First nucleotide	U (79%)	U (80%)
		Purine bias	**Yes**	**No**
	
	Excluded strand	First nucleotide	**C (76%)**	**C (37%) G (27%)**
		Pyrimidine bias	**Yes**	**No**
		GC basepair	**25/33 (76%)**	**15/30 (50%)**

In contrast, we find no specific sequence features characteristic of the miRNA pairs originating from the 103 precursor duplexes with an expression difference between the strands less than 10-fold (One-sided Wilcoxon test, *p *> 0.5 in all comparisons) (see Figures 2 and 3). Further, when the entire range of the expression differences within miRNA pairs is considered, rather than only the two extreme groups, we find a gradual increase in the 5'-nucleotide preference and the purine/pyrimidine content bias as the expression difference within pairs increases (see Additional file 1: Figure S3). This result was not caused by low expression of the miRNA pairs, as restricting the analysis to 50 miRNA pairs with each miRNA represented by more than 10 sequence reads did not affect the result.

To test reproducibility of this finding, we examined miRNA sequences in a technical replicate of the pooled cerebral cortical sample, as well as in a cortical sample from a single adult human male (see Methods). In both cases, we can identify the same sequence features characterising high- and low-expressed strands from precursors with a large strand selection bias, but not strands from precursors showing little bias (see Additional file 1: Figures S4 and S5). Further, all three features can be reproduced in another four samples representing other tissues and species: in the human cell line (HeLa) [26], human embryonic stem cells [27], human embryoid bodies [27], and mouse embryonic stem cells [28] (see Methods and Additional file 1: Table S4). Thus, all sequence features we find associated with miRNA strand selection in human brain are common among mammalian tissues.

Although reproducible, the observed sequence features might potentially be explained by factors other than strand selection bias within miRNA pairs. First, it is conceivable that Illumina sequencing itself is biased towards RNAs with a 5'-terminal U and a high purine content, and discriminates against RNAs with a 5'-terminal C and a high pyrimidine content. After examining all 3,650 unique sequences that can be mapped to the precursor region of known human miRNAs, we find neither a purine/pyrimidine bias, nor a terminal U/C bias (two sided Binomial test, *p *> 0.5) (see Additional file 1: Figure S6).

Second, the identified sequence features could reflect a common origin for the 33 examined miRNA pairs within the miRNA families. To assess this possibility, we retained only one randomly chosen miRNA pair to represent a family. After doing so, we still observe all three sequence features among the remaining 20 miRNA pairs (*p *< 0.05 in all tests).

Third, the observed sequence features could be associated with the high expression level of miRNAs, rather than with strand selection bias. Indeed, one of the miRNAs within a pair must be highly expressed to produce a greater than 1000-fold expression level difference. To assess this bias, we analyzed the sequence composition of 10 miRNA pairs with an expression level higher than 925 copies for at least one of the two miRNAs, and an expression difference within the pairs less than 10. Similarly, we sub-sampled 10 of the 33 miRNA pairs with an expression difference greater than 1,000-fold, while retaining a similar copy number distribution (see Additional file 1: Table S3). We find that while all three sequence features are still clearly observable for the 10 miRNA pairs with a large strand selection bias (*p *< 0.01 in all tests), none of the features are present among the 10 high-expressed miRNA pairs with little strand selection bias (*p *> 0.4 in all tests). Taken together, these results indicate that the identified sequence features are likely to be associated with strand selection bias within the human miRNA pairs.

### Strand selection determinants of fly miRNAs

To test whether the same sequence features determine expression differences within miRNA pairs in another organism, we used published data containing 2,010,618 Roche/454 sequence reads corresponding to 702,945 unique sequences collected from fruit fly (*Drosophila melanogaster*) S2 cells and from three tissues at 6 developmental stages [29]. After mapping these reads to the fly genome, we find sequences corresponding to 138 known fly miRNAs with a copy number equal or greater than two (see Methods). In addition, we identify 112 novel counterparts of known miRNAs (see Figure 1B). Thus, in flies as well as in humans, most miRNA precursors produce two expressed mature miRNA sequences.

We then determined whether specific sequence features determine strand selection bias in fly miRNA pairs. Since the fly sequence data has a smaller number of reads, we used expression differences greater than 30 as the strand selection bias cutoff for pairs, resulting in 30 miRNA pairs -- comparable to the number used in the human data analysis. Despite the smaller magnitude of expression differences within these miRNA pairs, we find a significant preference for the 5'-terminal U in the high-expressed duplex strand when compared to all expressed miRNAs (One-sided Binomial test, *p *= 0.0011). As in humans, the preference for a terminal uridine increases as the expression difference in miRNA pairs increases (from 76% to 85%). In contrast to humans, however, we find a preference for both C and G at the 5'-end of the low-expressed strand (One-sided Binomial test, *p *= 0.012 and *p *= 0.0078, respectively). Further, we find no detectable bias in the purine/pyrimidine composition between the high- and the low-expressed strands within 30 miRNA pairs (Wilcoxon test, *p *> 0.5) (see Table 1). Analyzing an additional *D. melanogaster *dataset containing sequences from 14 small RNA libraries and processed using Illumina platform [30], we obtain almost identical results (see Additional file 1: Table S5). Thus, sequence elements associated with preferential strand selection in humans and flies are not the same.

### Strand selection bias and miRNA processing

Can these additional sequence features found in humans facilitate asymmetric strand incorporation into an RISC? The presence of specific nucleotides on the 5'-end of both selected and excluded miRNAs is the first feature. In the plant *Arabidopsis thaliana*, the 5'-terminal nucleotides determine the sorting of various small RNAs into different Argonaute protein complexes [31]. It is intriguing to speculate that despite the vast differences between RNAi systems in humans and plants, human Argonaute proteins may have retained the ability to discriminate 5'-terminal nucleotides, even though for a different purpose.

Another feature is the preference for purine nucleotides in the high-expressed strand. We speculate that purine residues may facilitate strand loading into the RISC through sequence-independent interactions. Intriguingly, the PAZ domain of Argonaute proteins, which is involved in the initial steps of RNA recognition and binding, contains a large number of invariant aromatic residues involved in RNA binding [32-34]. It is therefore conceivable that the stacking and hydrophobic interactions between these residues and the purine-rich strand of the miRNA precursor duplex may contribute to strand selection.

Finally, as strand selection specificity can result in enormous expression level differences within some miRNA pairs, which in extreme cases can exceed one million copies, it is tempting to speculate that some of the sequence features we find may actively target the excluded miRNA strand for degradation. It is important to mention, however, that sequence features identified here show significant associated with strand selection bias, but do not explain all strand selection preferences found among miRNAs. Notably, recent observation that strand selection preferences may vary among tissues implies that strand selection factors other that sequence features might exist [19].

### Strand selection bias and miRNA-based regulation

Next we asked why strand selection bias exists for some miRNA pairs and, conversely, why some miRNA pairs are not biased. In our dataset, 15 of the 20 highest expressed miRNAs have a greater than 1000-fold expression difference between high- and low-expressed miRNA, while 19 showed a greater than 100-fold difference. Similarly, among the 100 most highly expressed miRNAs, 33 and 70 have a greater than 1000-fold and 100-fold expression difference, respectively. Thus, a large proportion of highly expressed miRNA precursors tends to generate only one highly expressed mature miRNA. This is intuitively understandable, since in the absence of strand selection bias each precursor would generate a pair of highly expressed mature miRNAs, collectively affecting the expression of the two groups of target genes. This condition must impose a strong constraint on the expression of an miRNA pair, as two different groups of target genes would have to be regulated in a coordinated fashion among various tissues and developmental stages.

If strand selection bias serves to limit the influence of a second mature miRNA on gene regulation, we expect to find fewer target genes for the low-expressed miRNA strand as compared to high-expressed one. Using the TargetScanS algorithm to identify evolutionarily conserved target sites [35], we indeed find that the high-expressed miRNAs have significantly more potential target genes than their low-expressed counterparts (One-sided Wilcoxon test, *p *= 0.0000013). This result confirms the notion that strand selection bias limits the regulatory role of a second mature miRNA that can be generated from a highly expressed precursor. It also indicates that the strand selection bias observed in the examined dataset is likely to persist throughout various tissues and various evolutionary lineages of vertebrates.

In contrast to the 33 miRNA pairs exhibiting a large strand selection bias, we find no significant difference in the target gene numbers between the high- and low-expressed miRNAs originating from the 103 pairs showing little bias (One-sided Wilcoxon test, *p *= 0.09). Further, the numbers of target genes for these miRNAs were significantly lower on average than the corresponding numbers for the high-expressed miRNAs from the 33 biased pairs (One-sided Wilcoxon test, *p *= 0.00001). Thus, expression of a single miRNA per precursor may be more compatible with the regulation of a large number of target genes due to lesser constraints imposed by the second miRNA. In fact, many of these miRNAs, such as the let-7 family, mir-29 family, mir-26 family, mir-140-3p, mir-30d, mir-7, mir-191, mir-21, mir-100, mir-15a, and mir-16, play an important role in development and differentiation [36-41].

### Strand selection and precursor hairpin arms bias

Finally, we asked whether highly expressed miRNAs preferentially originate from the 5p or 3p strand of the hairpin precursor. In humans we indeed find such a bias: highly expressed miRNAs originate more frequently from the 5p strand of the hairpin precursor for both the 33 biased miRNA pairs and for all expressed miRNAs (One-sided Binomial test, *p *= 0.0046 and *p *= 0.0017, respectively). In fruit flies, however, we find no such tendency (One-sided Binomial test, *p *= 0.67).

**Figure 4:**
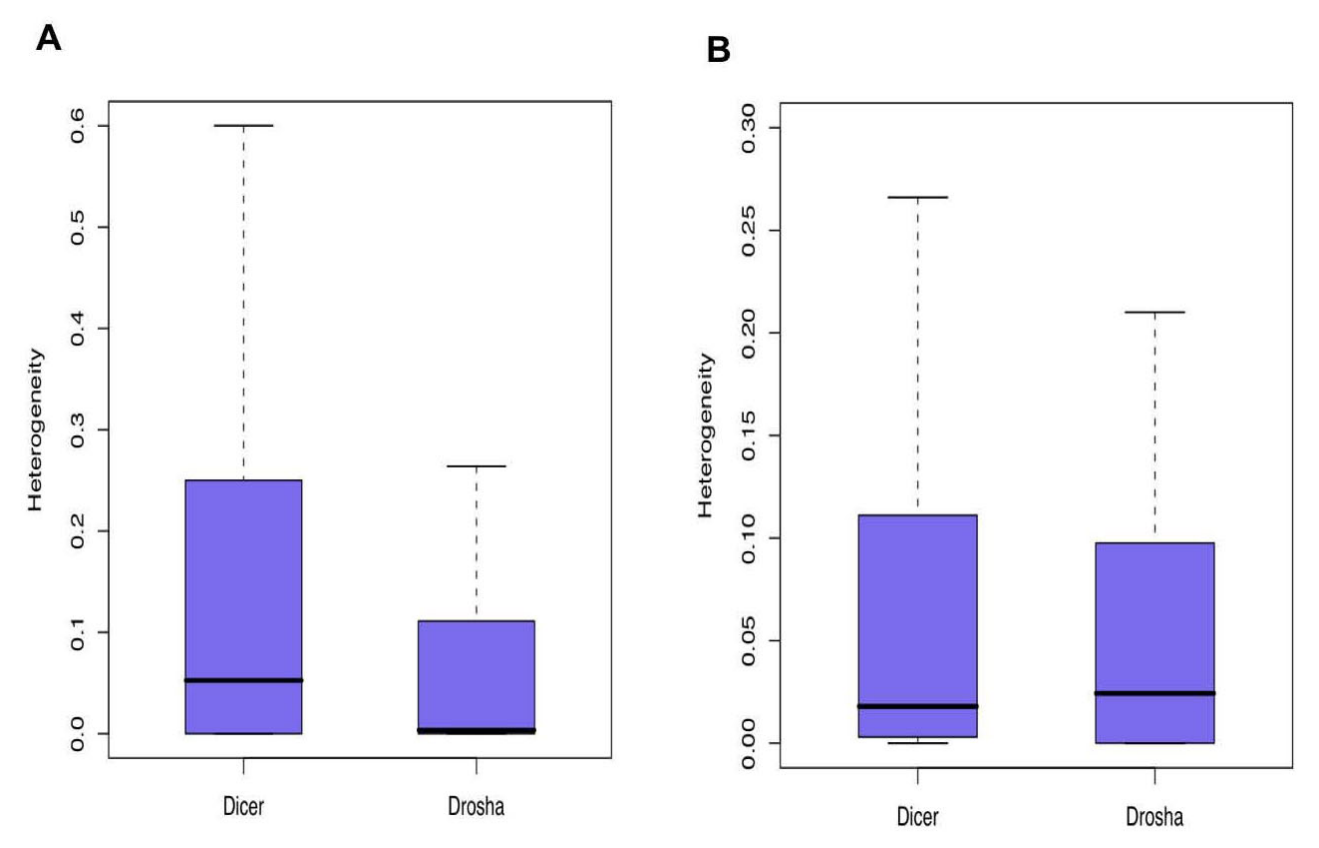
**miRNA 5'-end cutting accuracy comparison between Drosha and Dicer in humans and flies**. The bars show the mean value of 5'-end heterogeneity mature miRNAs defined by Dicer (left bar) or Drosha (right bar) in humans (Figure 4**A**) and flies (Figure 4**B**). For each miRNA, the heterogeneity was calculated as described in Methods. In the Figure 4A and Figure 4B, the Y-axis is the heterogeneity value, the top, middle and bottom lines of each box correspond to the first quartile, median, and the third quartile of observations, respectively. The vertical lines extend to the most extreme data points within 1.5 times the range between the first and the third quartiles. Data points outside this range are not shown.

Why would such a bias exist? One distinction between the two precursor arms is that the 5'-end of the miRNAs originating from the 5p hairpin arm is determined by Drosha, while for the 3p arm it is determined by Dicer. Since it is the 5'-end of a miRNA that determines its target specificity, incorrect cleavage may result in an accumulation of aberrant miRNA molecules. Indeed, during miRNA maturation, the 5'-end is cut more precisely than the 3'-end ([42,43] and our dataset, One-sided Wilcoxon test, *p *= 2.2*10^-16^). Still, if present in sufficiently high numbers, miRNAs with an aberrant 5'-end are likely to have a detrimental effect. In humans, we find highly expressed miRNAs to be biased towards the 5p hairpin arm. Based on this observation, we speculate that the cutting accuracy of the 5p-specific enzyme, Drosha, might be higher than that of Dicer. Indeed, we find that significantly fewer miRNA sequences with an incorrect 5'-end originate from the 5p precursor than from the 3p precursor strand in humans (One-sided Wilcoxon test, *p *= 0.000024) (see Figure 4A). We obtain the same result excluding all miRNA precursors containing possible alternative 5'-end isoforms [44] (One-sided Wilcoxon test, *p *= 1.99e-05) (see Methods). Other factors, such at non-template nucleotide addition or RNA editing may influence heterogeneity of miRNA ends. Still, we do not expect these factors to differ in effect between 5p and 3p strands of miRNA precursors. In contrast to humans, we did not observe any bias of highly expressed miRNAs towards the 5p or 3p precursor strand in flies. In agreement with our hypothesis, in flies we also do not find any significant differences in miRNA 5'-end cutting accuracy between the 5p and 3p hairpin arms (One-sided Wilcoxon test, *p *= 0.30) (see Figure 4B and Additional file 1: Table S5). This finding highlights another variation in miRNA maturation machinery between humans and flies.

## Conclusion

In conclusion, analyzing sequence features underlying miRNA strand selection bias in humans and flies, we find several outstanding features characterising human, but not fly miRNAs. These features include a bias towards specific nucleotides at the 5-ends of both selected and excluded strands (U and C, respectively), and a pronounced purine/pyrimidine content difference between the two strands. We speculate that these additional sequence features may have evolved to facilitate discrimination between the two stands of the precursor duplex. Such a mechanism might be particularly important for highly expressed precursor duplexes, when specific selection of one miRNA species and elimination of the other one are required for efficient target gene regulation. Further, we identify another feature distinguishing human and fly miRNA processing machinery: the relative accuracy of the Drosha and Dicer enzymes. These findings indicate that human and fly miRNA processing pathways are more distinct than currently recognized. Further, as both accurate processing and correct selection of the miRNA strand are crucial for RNAi performance, the miRNA sequence features described here are instrumental for the rational design of efficient miRNA-based expression regulators.

## Methods

### Sample preparation and sequencing

Human tissue was obtained from the NICHD Brain and Tissue Bank for Developmental Disorders at the University of Maryland, Baltimore, MD. The role of the NICHD Brain and Tissue Bank is to distribute tissue, and therefore, cannot endorse the studies performed or the interpretation of results. Informed consent for use of the human tissues for research was obtained in writing from all donors or the next of kin. All subjects were defined as normal controls by forensic pathologists at the NICHD Brain and Tissue Bank. No subjects who suffered a prolonged agonal state were used. All samples were taken from the frontal part of the superior frontal gyrus: a cortical region approximately corresponding to Brodmann area 9. For all samples similar proportions of grey and white matter were dissected. Total RNA was isolated from the frozen prefrontal cortex tissue using the Trizol (Invitrogen, USA) protocol with no modifications. Prior to low molecular weight RNA isolation, the total RNA from 20 male individuals aged between 14 and 58 years was combined in equal amounts. Low molecular weight RNA was isolated, ligated to the adapters, amplified, and sequenced following the Small RNA preparation protocol (Illumina, USA) with no modifications. Technical replication was completed by independently processing the mixed sample of 20 individuals starting from the low molecular weight RNA isolation step. Biological replication was performed by isolating and sequencing low molecular weight RNAs from a single 25-year-old individual. All produced sequences are deposited at the .

### Mapping of sequence reads to the human genome

For each of the three brain sequencing datasets, as well as for hESCs, hEBs and HeLa cells dataset, to remove the adapter sequence at the 3'-end of the sequence reads, all unique sequences were trimmed using the custom trimming procedure. Specifically, we first filtered out the low compositional complexity sequences using the DUST algorithm [45]. The remaining sequences were trimmed by matching the adapter sequence to the 3'-end allowing 3 mismatches if the length of the match was greater than 10 nucleotides and allowing 2 mismatches if the length of the match was between 4 and 10 nucleotides. The trimmed sequences were mapped to the human genome (hg18) [46] using the Illumina-supplied algorithm, ELAND. Only sequences perfectly matching the genome and with a length ranging from 18 to 28 nucleotides were retained. Using these parameters, approximately 60% of total sequence reads of each sample could be mapped. Of these sequences, we expect less than 1% to map incorrectly, as determined by a mapping of scrambled reads with the same length and nucleotide content distribution.

### Quantification of known human miRNAs

Human miRNA annotations, including the sequences and genomic locations of miRNA precursors and mature miRNA sequences, were downloaded from miRBase version 11 [47-49]. The genomic location of each mature miRNA was then derived using these data. All mapped sequences with a copy number equal to or greater than two were used for the following quantification procedure. First, all sequences mapping within three nucleotides upstream or downstream of the annotated 5'-position of the mature miRNAs were retained. Then, for each mature miRNA, the sequence with a maximal copy number was designated as the reference sequence. Finally, the expression level of each miRNA was calculated as a sum of the copy number of the reference sequence and the sequences mapping at the same 5'-end position as the reference sequence. The expression measurements obtained using this method and using the annotated mature miRNA sequence as a reference were similar, but the former method resulted in a slightly better correlation between experimental replicates and was used throughout the analysis (data not shown). The only exception was for the analysis of 5' and 3' miRNA sequence heterogeneity, when all sequences mapping within five nucleotides of the annotated 5'-end were retained.

### Novel microRNA detection

For the miRNA precursors with one annotated miRNA, small sequences mapping to the opposite arm of the precursor hairpin were analysed. The sequence with the maximal copy number was considered as a novel miRNA candidate. A further criterion required the existence of at least 14 basepairs between an annotated miRNA and a novel miRNA candidate within the precursor hairpin. The quantification process for novel miRNAs was the same as for known miRNAs.

### Mapping fly sequencing data to the Drosophila melanogaster genome

Roche/454 and Solexa sequences of the *Drosophila melanogaster *small RNA libraries were downloaded from GEO [GSE7448] [29] and [GSE11624] [50], and mapped to the *Drosophila melanogaster *genome (dm3, BDGP Release 5 from UCSC) using SOAP [51], respectively. Only sequences perfectly matching the genome and with a length ranging from 16 to 28 nt were retained. The minimal length cutoff was determined by mapping scrambled sequences to ensure that the proportion of falsely mapped reads was less than 1%. Detection of novel fly miRNAs and miRNA quantification were performed exactly as for human miRNAs.

### Mapping mouse sequencing data to mouse genome

Solexa sequences of mouse ESC small RNA library were downloaded from GEO [GSM314552] [28] and mapped to the mouse genome (mm9) using SOAP. Only sequences perfectly matching the genome and with a length ranging from 18 to 28 nt were retained. Detection of novel mouse miRNAs and miRNA quantification were performed exactly as for human miRNAs.

### miRNA target prediction

Potential miRNA target sites were determined using the TargetScanS algorithm [6,7,52]. TargetScanS predicts conserved targets of miRNAs by searching the 3'-UTR region of all annotated human genes for the presence of 7-mer and 8-mer sequences perfectly matching the miRNA seed region and conserved among 4 vertebrate species (human, mouse, rat, and dog).

### Sequence logo and statistical analyses

Sequence logos were generated using WebLogo [53]. Statistical analysis of the sequence features was performed using R [54]. Specifically, in the 5'-nucleotide prevalence analysis, the expected probability of the Binomial test was calculated based on the 5'-nucleotide base composition of all 847 annotated human miRNAs, plus 48 new miRNAs. For the purine/pyrimidine bias analysis, the nucleotide content of high- and low-expressed miRNAs was compared to the nucleotide content of high- and low-expressed miRNAs in all 253 expressed miRNA pairs and to the expressed miRNA pairs, excluding the tested group, using one-sided Wilcoxon test. Specifically, the one-sided Wilcoxon test results were: for 33 biased miRNA pairs (see Additional file 1: Table S1), when using all 253 pairs as one control, *p *= 0.013, *p *= 0.0011; using all 253 pairs and excluding the tested group (33 biased miRNAs) as one control, *p *= 0.0011, *p *= 0.00023; for 103 low-bias miRNA pairs (see Additional file 1: Table S2), using all 253 pairs as one control, *p *= 0.55, *p *= 0.56; and using all 253 pairs and excluding the tested group (low-bias miRNA pairs) as one control, *p *= 0.65, *p *= 0.64. (see Figures 2, 3 and Additional file 1: Figure S7)

For the 10 biased and 10 low-bias high-expressed miRNA pairs (see Additional file 1: Table S3), the purine/pyrimidine bias analysis was performed by comparing the purine content and pyrimidine content of the high- and low-expressed miRNAs within the pairs, due to the small sample size. The one-sided Wilcoxon test results were: for purine enrichment of 10 biased miRNA pairs, *p *= 0.00015; for 10 low-bias miRNA pairs, *p *= 0.44.

For HeLa data, the statistical results were as follows: preference for U and C at the 5'-ends of high- and low-expressed miRNAs, respectively (One-sided Binomial test, *p *= 8.6+10^-7 ^and *p *= 7.2+10^-8^) and purine/pyrimidine bias (One sided Wilcoxon test, *p *< 0.05). (see Additional file 1: Table S4)

For hESCs data, the statistical results were as follows: preference for U and C at the 5'-ends of high- and low-expressed miRNAs, respectively (One-sided Binomial test, *p *= 1.8+10^-4 ^and *p *= 3.2+10^-7^) and purine/pyrimidine bias (One sided Wilcoxon test, *p *< 0.05). (see Additional file 1: Table S4)

For hEBs data, the statistical results were as follows: preference for U and C at the 5'-ends of high- and low-expressed miRNAs, respectively (One-sided Binomial test, *p *= 1.7+10^-5 ^and *p *= 4.3+10^-8^) and purine/pyrimidine bias (One sided Wilcoxon test, *p *< 0.05). (see Additional file 1: Table S4)

For Mouse ESC cells data, the statistical results were as follows: preference for U and C at the 5'-ends of high- and low-expressed miRNAs, respectively (One-sided Binomial test, *p *= 0.008185 and *p *= 3.664e+10^-5^) and purine/pyrimidine bias (One sided Wilcoxon test, *p *< 0.05). (see Additional file 1: Table S4)

### Dicer and Drosha cutting accuracy analysis

For each miRNA, the heterogeneity of its termini was calculated as the method described by [42].

Briefly, first, all sequences mapping within five nucleotides upstream or downstream of the annotated 5'-position of the mature miRNAs were retained. Then, for each mature miRNA, the sequence with a maximal copy number was designated as the reference sequence. Finally, the heterogeneity of its termini was calculated as the mean of the absolute distances between the observed 5'- or 3'- ends and the ends of the reference sequence. The 5'-cutting accuracy of Dicer and Drosha was compared using Wilcoxon test. (see Figure 4A and 4B).

To exclude the influence of the alternative precursor cleavage by Drosha and Dicer that may influence miRNA end heterogeneity estimate, we remove the miRNA precursors potentially containing alternative cleavage isoforms. We judged whether a miRNA have an alternative cleavage isoform by checking whether the expression of the second most expressed isoform takes up more than 30% of the expression of the most expressed one. Using such a criterion, 16 miRNAs expressed in the human brain dataset can be judged to undergo alternative cleavage. 7 out of 16 originate from the 5' arm, which 5'end is defined by Drosha, while 9 come from the 3' arm, which 5'end is defined by Dicer. After excluding these miRNAs from the analysis, we still find that the cutting accuracy of Drosha at the miRNA 5'end is significantly higher than the cutting accuracy of Dicer (One side Wilcox test p-value = 1.99e-05).

## Authors' contributions

ZY and CM carried out the experiments, HYH, YX, and HH analyzed the data, HYH, WC, and PK drafted the manuscript. YHZ, WC, and PK conceived of the study, and participated in its design and coordination. All authors read and approved the final manuscript.

## Supplementary Material

Additional file 1 **Supplemental Supporting information of figures and tables**. The following additional data are available with the online version of this paper. Additional data file 1 contains all supplementary figures, tables and related descriptions. Click here for file
