# Supplementary material for: Sequence features associated with microRNA strand selection in humans and flies
Source: BMC Genomics. 2009 Sep 4;10:413. doi: 10.1186/1471-2164-10-413 (PMC2751786; doi:10.1186/1471-2164-10-413)
Supplement: Additional file 1 — Supplemental Supporting information of figures and tables. The following additional data are available with the online version of this paper. Additional data file 1 contains all supplementary figures, tables and related descriptions. [file 1471-2164-10-413-S1.doc]

**Supporting information for** “**Sequence features associated with microRNA strand selection in humans and flies”**

**Supplementary Figures**

**
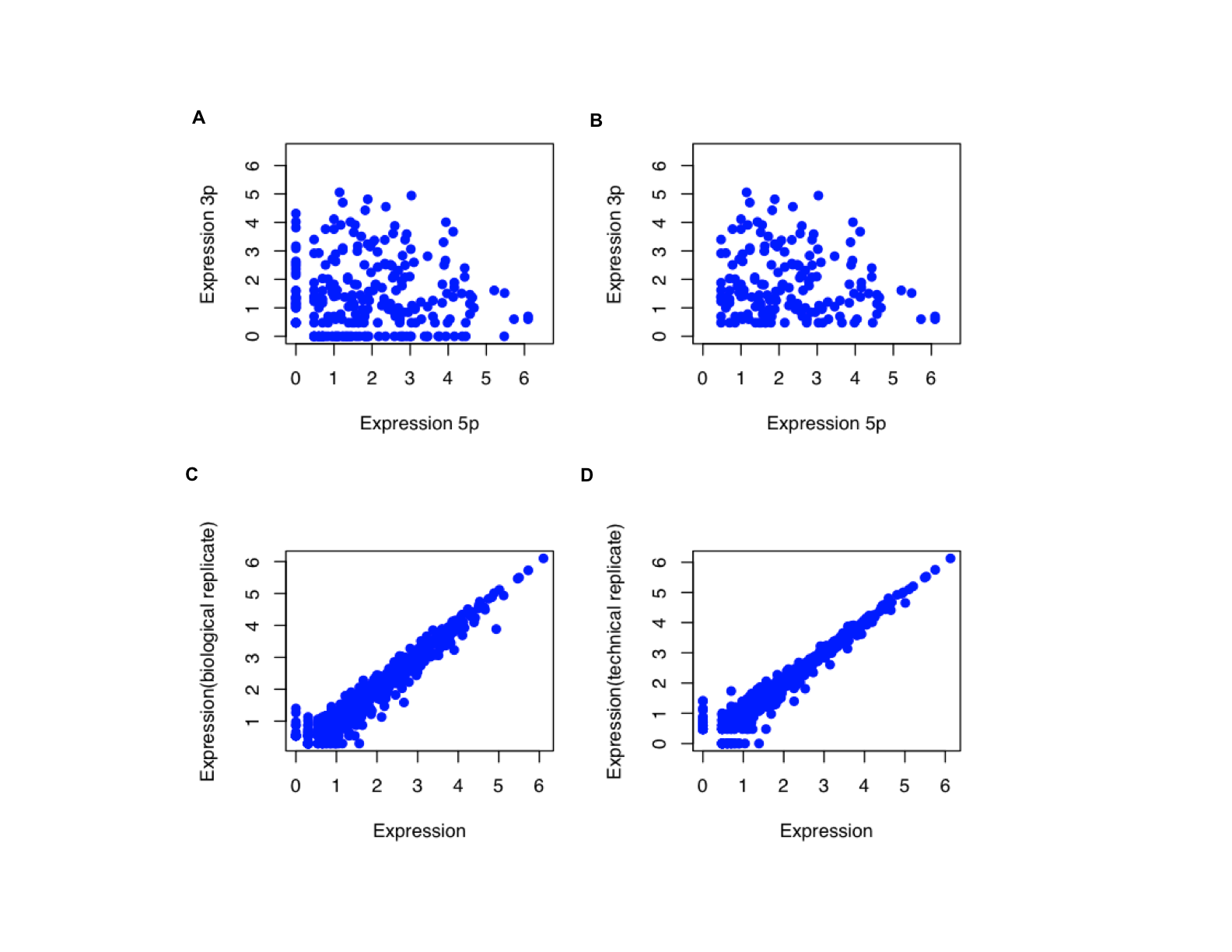
**

**Figure S1.** **miRNA expression correlation.** (**A**) Expression levels of human mature miRNAs originating from 5p (x-axis) or 3p (y-axis) arms of the hairpin precursor. Expression levels are plotted on log-10 scale in all panels. The plot includes annotated miRNA sequences with no detectable expression in our dataset. (**B**) The same as (**A)**, but excluding annotated miRNA sequences with no detectable expression. (**C**) and (**D**) show miRNA expression levels in the pooled sample studied and its biological and technical replicates, respectively.

**
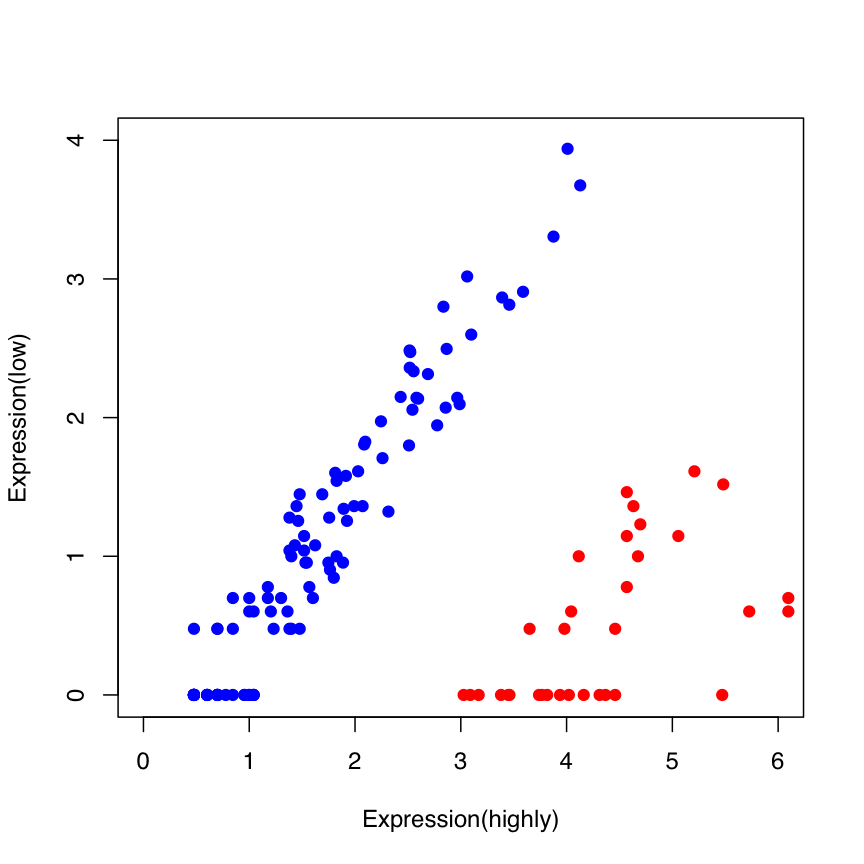
**

**Figure S2. Expression levels of human miRNA pairs.** Expression levels of high- and low-expressed strands from 33 miRNAs pairs with large stand selection bias (red) and 103 miRNAs pairs with little stand selection bias (blue). The expression levels are plotted on the log-10 scale. See Tables S1 and S2 for complete information.


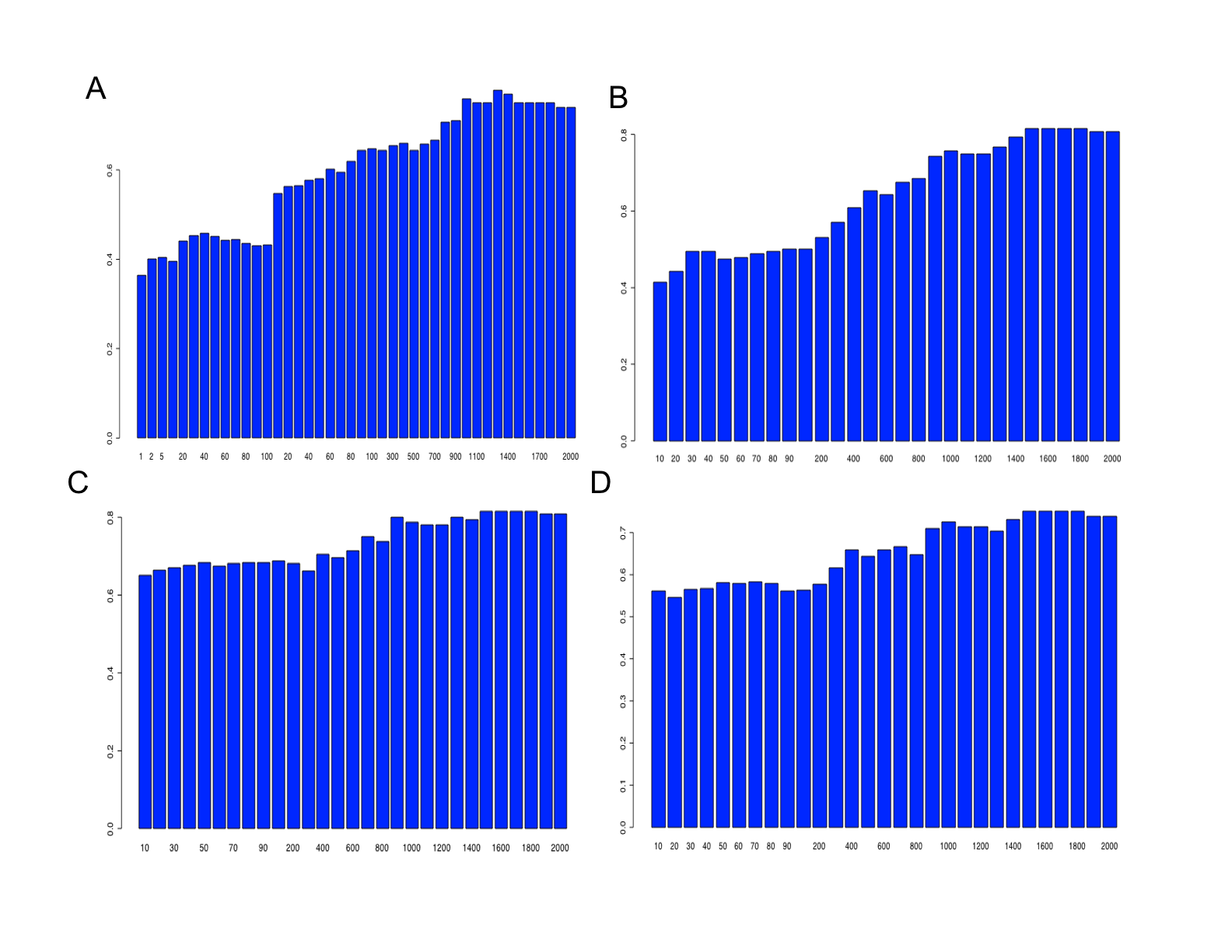


**Figure S3. Changes in the 5’-nucleotide preference and the purine/pyrimidine content depending on strand selection bias.** (**A**) Proportion of U at the 5’-position high-expressed miRNA strand (y-axis) versus the expression ratio between high- and low-expressed miRNA strands within miRNA pairs (x-axis). The x-axis is the same for all four panels. (**B**) Proportion of C at the 5’-position low-expressed miRNA strand

(**C**) Proportion of miRNA which purine content larger than 50% in the high-expressed miRNA strand. (**D**) Proportion of miRNA which pyrimidine content larger than 50% in the low-expressed miRNA strand.


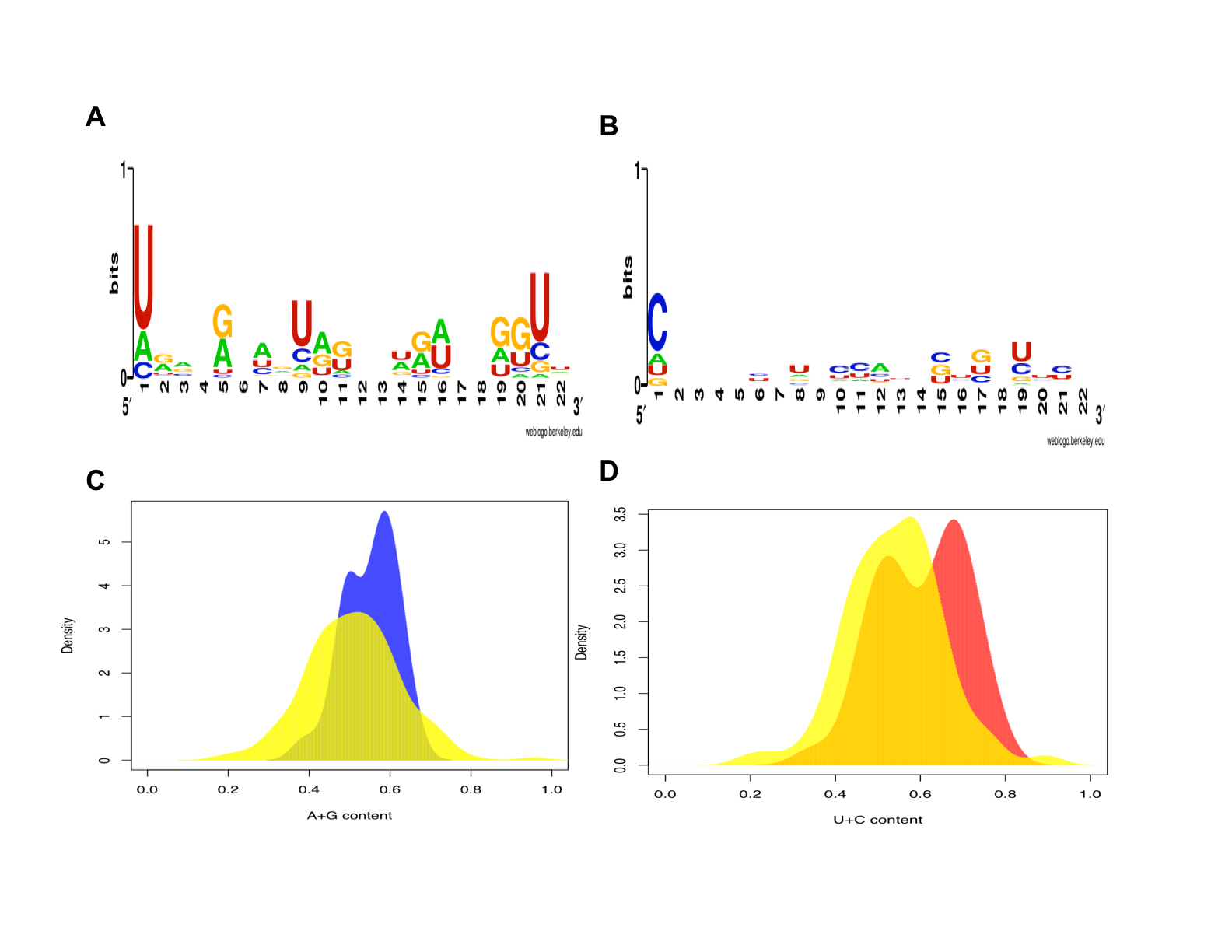


**Figure S4. Sequence features characteristic to miRNA pairs with large strand selection bias can be reproduced in a technical replicate.** Technical replicate data was produced by independent sample preparation and sequencing starting from a shared total miRNA sample. Sequence composition of high- expressed (**A**) and low-expressed (**B**) strands from 33 miRNA pairs with large strand selection bias. (**C**) Purine content of the high-expressed strands from 33 miRNA pairs with large strand selection bias (blue) and from all other expressed miRNA pairs (yellow). (**D**) Pyrimidine content of the low-expressed strands from 33 miRNA pairs with large strand selection bias (red) and from all other expressed miRNA pairs (yellow).


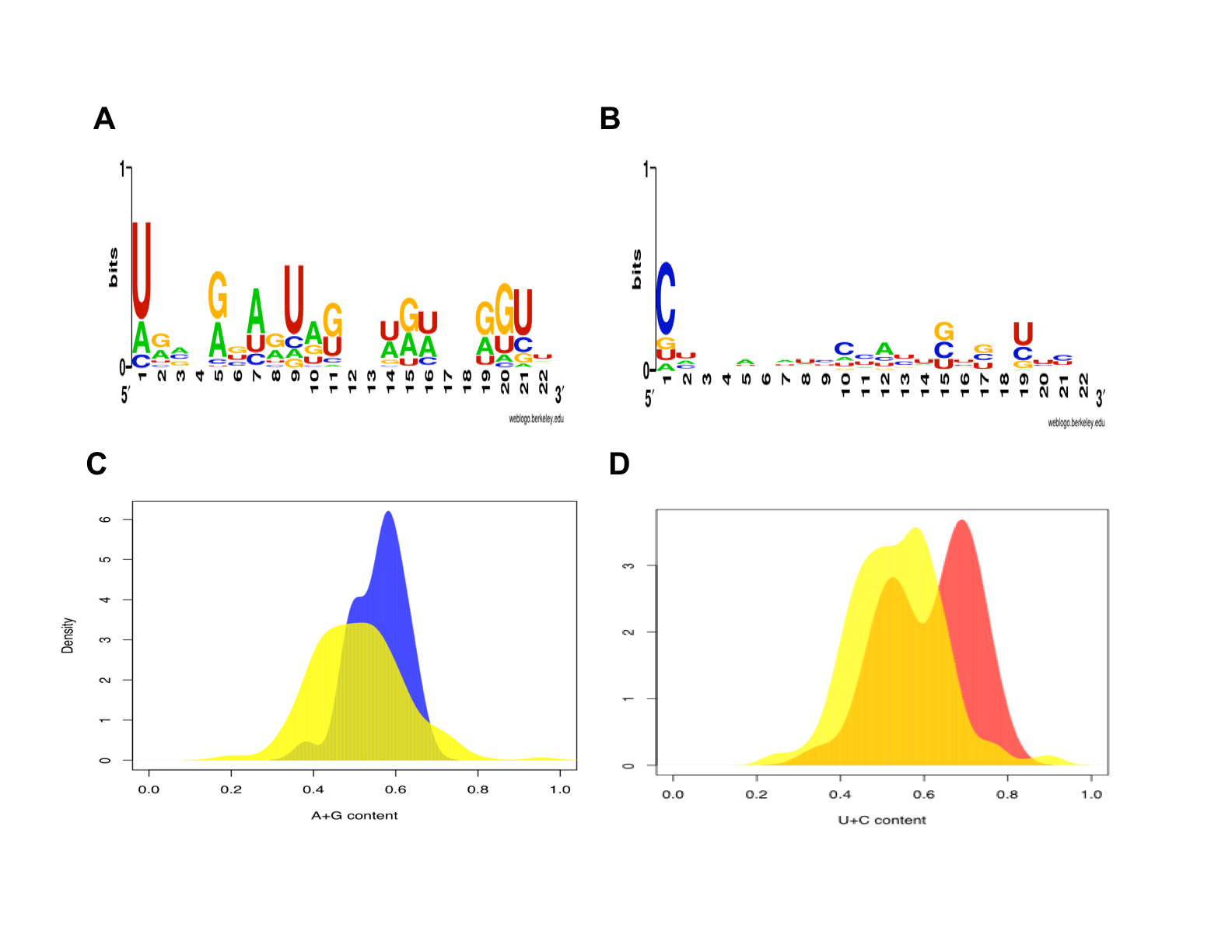


**Figure S5. Sequence features characteristic to miRNA pairs with large strand selection bias can be reproduced in a biological replicate.** Biological replicate data was produced by independent sample preparation and sequencing using an independent sample from a single adult male human. Panel information is as on Figure S4.


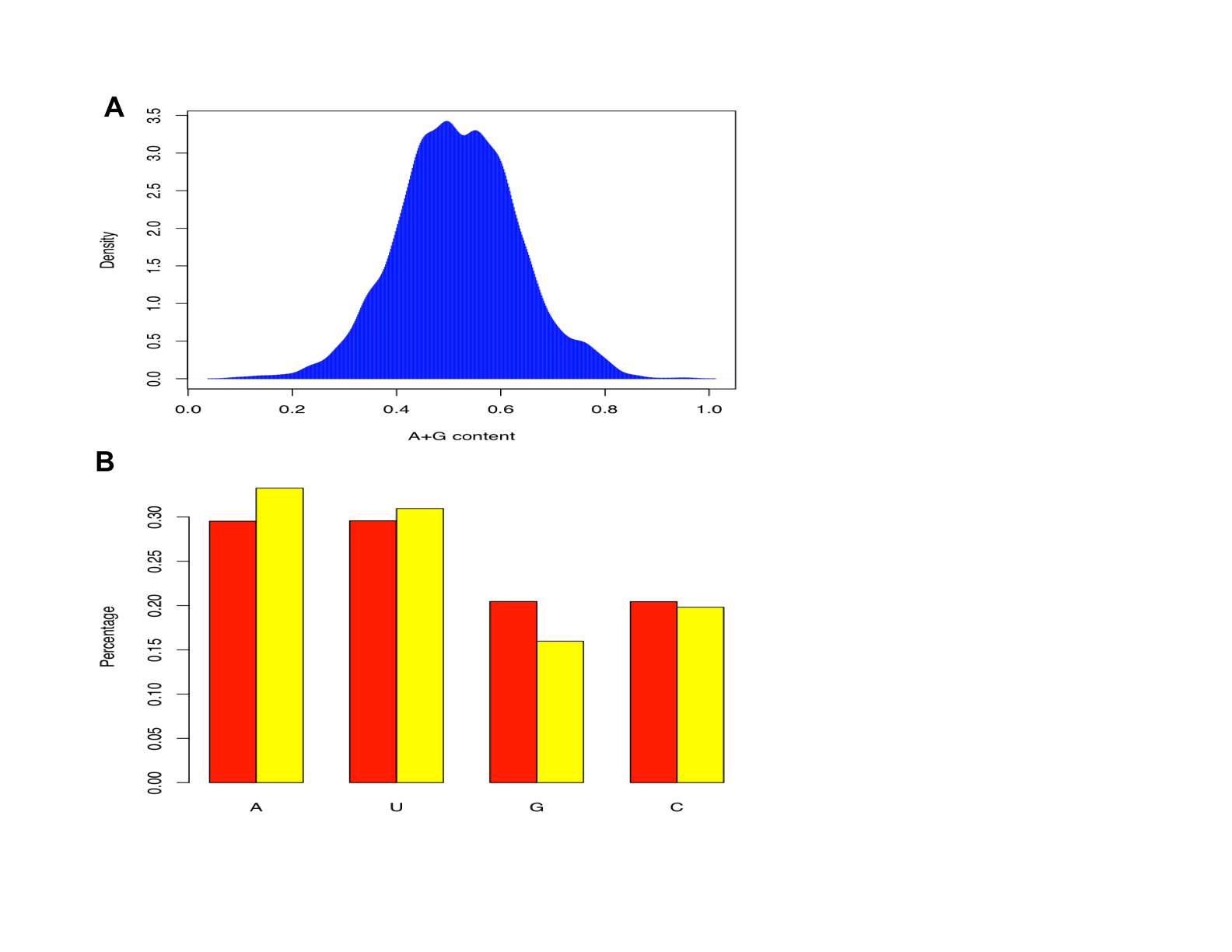


**Figure S6. Purine/pyrimidine content and the first nucleotide identity in illumina sequences.** Shown are purine/pyrimidine content (**A**) and the 5’ nucleotide identity (**B**) of all 3,650 unique sequences that can be mapped to the precursor region of known human miRNAs. The red and the yellow bars represent nucleotide frequencies in the human genome and at the 5’-position in 3,650 unique sequences, respectively.

**
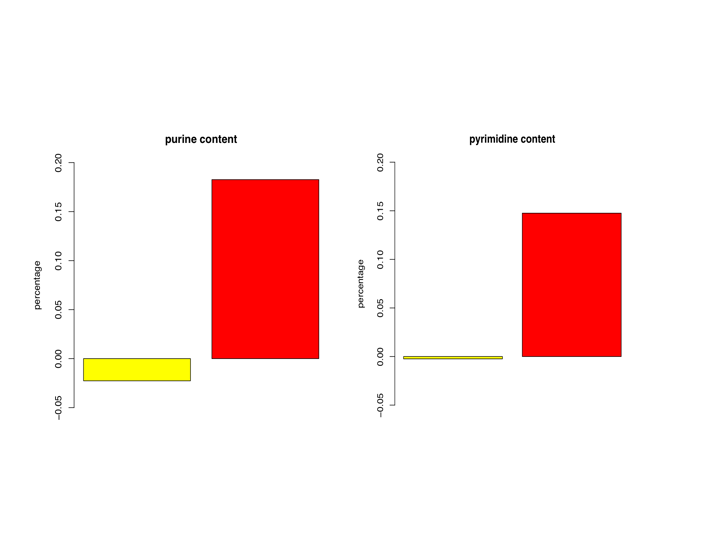
**

**Figure S7. Purine/Pyrimidine content bias of 33 miRNA pairs with large strand selection bias**

The difference in proportion of miRNAs with excess (>50%) of purines in high-expressed miRNA strand (**A**) and pyrimidines in low expressed miRNA strand (**B**) in 33 miRNA pairs with large strand selection bias (right column, red) and from 103 miRNA pairs with little strand selection bias (left column, yellow) compared to all high-expressed and low-expressed miRNAs, respectively.

**Supplementary tables**

**Table S1.** Human miRNAs from 33 miRNA pairs with large strand selection bias

| ***miRNA_id^a^*** | ***Expression*** | ***miRNA_id^b^*** | ***Expression*** |
| --- | --- | --- | --- |
| hsa-let-7f | 1248918 | hsa-let-7f-1* | 4 |
| hsa-let-7f | 1248918 | hsa-let-7f-2* | 3 |
| hsa-let-7g | 533067 | hsa-let-7g* | 3 |
| hsa-let-7a | 302759 | hsa-let-7a* | 32 |
| hsa-let-7c | 296128 | hsa-let-7c* | 0 |
| hsa-let-7b | 161662 | hsa-let-7b* | 40 |
| hsa-mir-29a | 113936 | hsa-mir-29a* | 13 |
| hsa-mir-140-3p | 49819 | hsa-mir-140-5p | 16 |
| hsa-let-7i | 47324 | hsa-let-7i* | 9 |
| hsa-let-7e | 42845 | hsa-let-7e* | 22 |
| hsa-mir-7 | 37118 | hsa-mir-7-2* | 5 |
| hsa-mir-7 | 37118 | hsa-mir-7-3-3p | 28 |
| hsa-mir-7 | 37118 | hsa-mir-7-1* | 13 |
| hsa-mir-26a | 28799 | hsa-mir-26a-2* | 2 |
| hsa-mir-26a | 28799 | hsa-mir-26a-1* | 0 |
| hsa-mir-340 | 23312 | hsa-mir-340* | 0 |
| hsa-mir-101 | 20515 | hsa-mir-101* | 0 |
| hsa-mir-26b | 14531 | hsa-mir-26b* | 0 |
| hsa-mir-29c | 13035 | hsa-mir-29c* | 9 |
| hsa-mir-191 | 11051 | hsa-mir-191* | 3 |
| hsa-mir-222 | 10511 | hsa-mir-222* | 0 |
| hsa-mir-34c-5p | 9545 | hsa-mir-34c-3p | 2 |
| hsa-mir-21 | 8667 | hsa-mir-21* | 0 |
| hsa-mir-378 | 6554 | hsa-mir-378* | 0 |
| hsa-mir-100 | 5844 | hsa-mir-100* | 0 |
| hsa-mir-192 | 5490 | hsa-mir-192* | 0 |
| hsa-mir-30d | 4471 | hsa-mir-30d* | 2 |
| hsa-mir-16 | 2884 | hsa-mir-16-1* | 0 |
| hsa-mir-432 | 2817 | hsa-mir-432* | 0 |
| hsa-mir-744 | 2399 | hsa-mir-744* | 0 |
| hsa-mir-29b | 1475 | hsa-mir-29b-1* | 0 |
| hsa-mir-130a | 1227 | hsa-mir-130a* | 0 |
| hsa-mir-15a | 1063 | hsa-mir-15a* | 0 |

^a^ miRNAs corresponding to high-expressed strand

^b^ miRNAs corresponding to low-expressed strand

**Table S2.** Human miRNAs from 103 miRNA pairs with little strand selection bias

| **miRNA_id^a^** | **Expression** | **miRNA_id^b^** | **Expression** |
| --- | --- | --- | --- |
|  |  |  |  |
| hsa-mir-9 | 13457 | hsa-mir-9* | 4729 |
| hsa-mir-221 | 10220 | hsa-mir-221* | 8686 |
| hsa-mir-485-5p | 7523 | hsa-mir-485-3p | 2020 |
| hsa-mir-151-3p | 3878 | hsa-mir-151-5p | 807 |
| hsa-mir-423-5p | 2875 | hsa-mir-423-3p | 651 |
| hsa-mir-129-3p | 2458 | hsa-mir-129-5p | 734 |
| hsa-mir-30e | 1254 | hsa-mir-30e* | 396 |
| hsa-mir-382-3p | 1148 | hsa-mir-382 | 1041 |
| hsa-mir-212-5p | 974 | hsa-mir-212 | 124 |
| hsa-mir-338-3p | 925 | hsa-mir-338-5p | 138 |
| hsa-mir-129-5p | 734 | hsa-mir-129* | 312 |
| hsa-mir-106b | 719 | hsa-mir-106b* | 117 |
| hsa-mir-23b | 684 | hsa-mir-23b* | 630 |
| hsa-mir-708 | 597 | hsa-mir-708* | 87 |
| hsa-mir-30c | 488 | hsa-mir-30c-2* | 205 |
| hsa-mir-1307-5p | 394 | hsa-mir-1307 | 136 |
| hsa-mir-361-5p | 380 | hsa-mir-361-3p | 138 |
| hsa-mir-132 | 357 | hsa-mir-132* | 215 |
| hsa-mir-181c | 348 | hsa-mir-181c* | 113 |
| hsa-mir-374a | 332 | hsa-mir-374a* | 296 |
| hsa-mir-409-3p | 328 | hsa-mir-409-5p | 228 |
| hsa-mir-17* | 327 | hsa-mir-17 | 304 |
| hsa-mir-135a-3p | 323 | hsa-mir-135a | 62 |
| hsa-mir-126 | 269 | hsa-mir-126* | 140 |
| hsa-mir-144* | 206 | hsa-mir-144 | 20 |
| hsa-mir-136 | 181 | hsa-mir-136* | 50 |
| hsa-mir-28-3p | 175 | hsa-mir-28-5p | 93 |
| hsa-mir-145 | 124 | hsa-mir-145* | 66 |
| hsa-mir-1185 | 121 | hsa-mir-1185-3p | 63 |
| hsa-mir-487a | 117 | hsa-mir-487a-5p | 22 |
| hsa-mir-369-5p | 106 | hsa-mir-369-3p | 40 |
| hsa-mir-490-3p | 97 | hsa-mir-490-5p | 22 |
| hsa-mir-193b* | 83 | hsa-mir-193b | 17 |
| hsa-mir-424 | 81 | hsa-mir-424* | 37 |
| hsa-mir-204 | 77 | hsa-mir-204-3p | 21 |
| hsa-mir-324-5p | 76 | hsa-mir-324-3p | 8 |
| hsa-mir-329 | 66 | hsa-mir-329-5p | 34 |
| hsa-mir-339-3p | 66 | hsa-mir-339-5p | 9 |
| hsa-mir-425* | 64 | hsa-mir-425 | 39 |
| hsa-mir-135a | 62 | hsa-mir-135a* | 6 |
| hsa-mir-154* | 57 | hsa-mir-154 | 7 |
| hsa-mir-193a-5p | 56 | hsa-mir-193a-3p | 18 |
| hsa-mir-1298 | 55 | hsa-mir-1298-3p | 8 |
| hsa-mir-365-5p | 48 | hsa-mir-365 | 27 |
| hsa-mir-299-3p | 41 | hsa-mir-299-5p | 11 |
| hsa-mir-1306 | 39 | hsa-mir-1306-5p | 4 |
| hsa-mir-380* | 36 | hsa-mir-380 | 5 |
| hsa-mir-381 | 34 | hsa-mir-381-5p | 8 |
| hsa-mir-874 | 33 | hsa-mir-874-5p | 8 |
| hsa-mir-20b | 32 | hsa-mir-20b* | 10 |
| hsa-mir-377* | 32 | hsa-mir-377 | 13 |
| hsa-mir-30b | 29 | hsa-mir-30b* | 27 |
| hsa-mir-505* | 29 | hsa-mir-505 | 2 |
| hsa-mir-654-5p | 28 | hsa-mir-654-3p | 17 |
| hsa-mir-625 | 27 | hsa-mir-625* | 22 |
| hsa-mir-223 | 26 | hsa-mir-223* | 11 |
| hsa-mir-582-3p | 24 | hsa-mir-582-5p | 9 |
| hsa-mir-331-3p | 24 | hsa-mir-331-5p | 2 |
| hsa-mir-876-3p | 23 | hsa-mir-876-5p | 18 |
| hsa-mir-34a | 23 | hsa-mir-34a* | 10 |
| hsa-mir-766-5p | 23 | hsa-mir-766 | 2 |
| hsa-mir-105* | 22 | hsa-mir-105 | 3 |
| hsa-mir-376a | 19 | hsa-mir-376a* | 4 |
| hsa-mir-496 | 16 | hsa-mir-496-5p | 2 |
| hsa-mir-641 | 15 | hsa-mir-641-3p | 3 |
| hsa-mir-576-3p | 14 | hsa-mir-576-5p | 4 |
| hsa-mir-671-5p | 14 | hsa-mir-671-3p | 5 |
| hsa-mir-516b | 10 | hsa-mir-516b* | 0 |
| hsa-mir-590-5p | 10 | hsa-mir-590-3p | 3 |
| hsa-mir-483-5p | 10 | hsa-mir-483-3p | 0 |
| hsa-mir-214 | 10 | hsa-mir-214* | 0 |
| hsa-mir-508-3p | 9 | hsa-mir-508-5p | 0 |
| hsa-mir-450a | 9 | hsa-mir-450a-3p | 4 |
| hsa-mir-455-3p | 9 | hsa-mir-455-5p | 3 |
| hsa-mir-200b | 9 | hsa-mir-200b* | 0 |
| hsa-mir-619-5p | 8 | hsa-mir-619 | 0 |
| hsa-mir-758-5p | 8 | hsa-mir-758 | 0 |
| hsa-mir-18a | 6 | hsa-mir-18a* | 2 |
| hsa-mir-544-5p | 6 | hsa-mir-544 | 4 |
| hsa-mir-32 | 6 | hsa-mir-32* | 0 |
| hsa-mir-1273-5p | 5 | hsa-mir-1273 | 0 |
| hsa-mir-296-5p | 4 | hsa-mir-296-3p | 0 |
| hsa-mir-362-5p | 4 | hsa-mir-362-3p | 2 |
| hsa-mir-1256-3p | 4 | hsa-mir-1256 | 2 |
| hsa-mir-10b | 4 | hsa-mir-10b* | 0 |
| hsa-mir-629 | 4 | hsa-mir-629* | 0 |
| hsa-mir-183 | 4 | hsa-mir-183* | 0 |
| hsa-mir-450b-5p | 4 | hsa-mir-450b-3p | 0 |
| hsa-mir-509-5p | 3 | hsa-mir-509-3p | 0 |
| hsa-mir-188-5p | 3 | hsa-mir-188-3p | 0 |
| hsa-mir-133a-5p | 3 | hsa-mir-133a | 0 |
| hsa-mir-541* | 3 | hsa-mir-541 | 0 |
| hsa-mir-548c-5p | 2 | hsa-mir-548c-3p | 0 |
| hsa-mir-599-5p | 2 | hsa-mir-599 | 0 |
| hsa-mir-605-3p | 2 | hsa-mir-605 | 0 |
| hsa-mir-371-5p | 2 | hsa-mir-371-3p | 0 |
| hsa-mir-454 | 2 | hsa-mir-454* | 0 |
| hsa-mir-597-3p | 2 | hsa-mir-597 | 0 |
| hsa-mir-548d-5p | 2 | hsa-mir-548d-3p | 0 |
| hsa-mir-770-5p | 2 | hsa-mir-770-5p-3p | 2 |
| hsa-mir-19a | 2 | hsa-mir-19a* | 0 |
| hsa-mir-519a* | 2 | hsa-mir-519a | 0 |
| hsa-mir-548b-3p | 2 | hsa-mir-548b-5p | 0 |

^a^ miRNAs corresponding to high-expressed strand

^b^ miRNAs corresponding to low-expressed strand

**Table S3.** Expression level of 10 highly expressed miRNAs from miRNA pairs with large and little strand selection bias

| miRNA_id^a^ | Expression | miRNA_id^b^ | Expression |
| --- | --- | --- | --- |
| hsa-mir-9 | 13457 | hsa-mir-29c | 13035 |
| hsa-mir-221 | 10220 | hsa-mir-222 | 10511 |
| hsa-mir-485-5p | 7523 | hsa-mir-21 | 8667 |
| hsa-mir-151-3p | 3878 | hsa-mir-30d | 4471 |
| hsa-mir-423-5p | 2875 | hsa-mir-16 | 2884 |
| hsa-mir-129-3p | 2458 | hsa-mir-432 | 2817 |
| hsa-mir-30e | 1254 | hsa-mir-744 | 2399 |
| hsa-mir-382-3p | 1148 | hsa-mir-29b | 1475 |
| hsa-mir-212-5p | 974 | hsa-mir-130a | 1227 |
| hsa-mir-338-3p | 925 | hsa-mir-15a | 1063 |

^a^ High-expressed miRNAs from miRNA pairs with little strand selection bias

^b^ High-expressed miRNAs from miRNA pairs with large strand selection bias

**Table S4. Sequence features associated with miRNA strand selection in four additional datasets**

|  | | hESCs^a^ | hEBs^b^ | Hela^c^ | mESCs^d^ |
| --- | --- | --- | --- | --- | --- |
| Selected Strand | 1^st^ nucleotide  (Percentage) | U (74%) | U (68%) | U (78%) | U (62%) |
|  | Purine bias  (One sided Wilcoxon test pvalue) | 0.001 | 0.005 | 0.039 | 0.013 |
| Exclude  Strand | 1^st^ nucleotide | C (68%) | C (65%) | C (64%) | C (50%) |
|  | Pyrimidine bias  (One sided Wilcoxon test pvalue) | 0.0002 | 0.0009 | 0.011 | 0.030 |

^a^ hESCs : Human embryonic stem cells

^b^ hEBs : Human embryoid bodies

^c^ Hela : Human Hela cell line

^d^ mESCs : Mouse embryonic stem cells

**Table S5.** Fruit fly miRNA sequence features comparison between two sequencing platforms

|  | | Solexa data | | 454 data |
| --- | --- | --- | --- | --- |
| Selected Strand | 1^st^ nucleotide  (Percentage) | U (84%) | | U (80%) |
|  | Purine bias | NO | | NO |
| Exclude Strand | 1^st^ nucleotide  (Percentage) | C (32%)  G (29%) | | C (37%)  G (27%) |
|  | Pyrimidine bias | NO | | NO |
| Cutting accuracy difference between Drosha and Dicer | | NO | NO | |

|  | |  |  |  |  |
| --- | --- | --- | --- | --- | --- |
|  |  |  |  |  |  |
|  |  |  |  |  |
|  |  |  |  |  |  |
|  |  |  |  |  |
